# Too many zeros and/or highly skewed? A tutorial on modelling health behaviour as count data with Poisson and negative binomial regression

**DOI:** 10.1080/21642850.2021.1920416

**Published:** 2021-05-06

**Authors:** James A. Green

**Affiliations:** a School of Allied Health, University of Limerick, Limerick, Ireland; b Physical Activity for Health Research Cluster (Health Research Institute), University of Limerick, Limerick, Ireland

**Keywords:** Count data, Poisson regression, negative binomial regression, skewed data, tutorial

## Abstract

**Background:** Dependent variables in health psychology are often counts, for example, of a behaviour or number of engagements with an intervention. These counts can be very strongly skewed, and/or contain large numbers of zeros as well as extreme outliers. For example, ‘How many cigarettes do you smoke on an average day?’ The modal answer may be zero but may range from 0 to 40+. The same can be true for minutes of moderate-to-vigorous physical activity. For some people, this may be near zero, but take on extreme values for someone training for a marathon. Typical analytical strategies for this data involve explicit (or implied) transformations (smoker v. non-smoker, log transformations). However, these data types are ‘counts’ (i.e. non-negative whole numbers) or quasi-counts (time is ratio but discrete minutes of activity could be analysed as a count), and can be modelled using count distributions – including the Poisson and negative binomial distribution (and their zero-inflated and hurdle extensions, which alloweven more zeros).

**Methods:** In this tutorial paper I demonstrate (in R, Jamovi, and SPSS) the easy application of these models to health psychology data, and their advantages over alternative ways of analysing this type of data using two datasets – one highly dispersed dependent variable (number of views on YouTube, and another with a large number of zeros (number of days on which symptoms were reported over a month).

**Results:** The negative binomial distribution had the best fit for the overdispersed number of views on YouTube. Negative binomial, and zero-inflated negative binomial were both good fits for the symptom data with over-abundant zeros.

**Conclusions:** In both cases, count distributions provided not just a better fit but would lead to different conclusions compared to the poorly fitting traditional regression/linear models.

Many measurements of health behaviours (and indeed, behaviour in general), are the number of times a person engages in that behaviour or the time spent on it. These numbers are counts – they are non-negative whole numbers (values below zero are not possible). This type of data is often not normal, very heavily skewed, and can have a few extremely high values that look like outliers but might still be credible values. And sometimes, but not always, there may be a lot of zeros. Some real examples are depicted in Figure 1.

**Figure 1.**
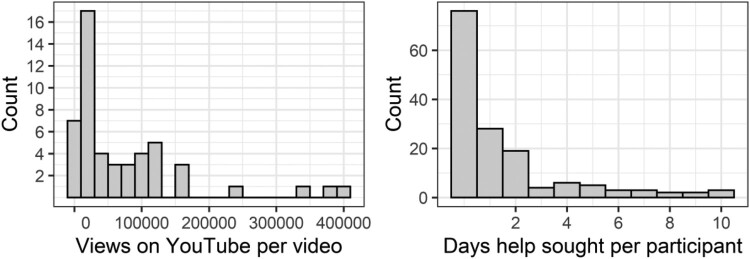
Histograms of real count data. Left panel is the number of views on YouTube of videos about scoliosis (Staunton et al., 2015). Right panel is the number of days on which participants sought help from a health professional over 30 days (Anwar et al., 2017).

These dependent variables are easily and accurately analysed with a class of models described as ‘count regressions’. These assume that rather than the dependent variable fitting a normal distribution – the bell curve – that the dependent variable fits a different class of distributions, all of which start at zero, and only take on whole integer values.

However, looking at the published literature, there are several less optimal approaches taken to dealing with this type of data. One common strategy is to dichotomise the data, especially where there are many zeros (Xie, Tao, McHugo, & Drake, 2013). The dependent variable then becomes those who engage in the behaviour (at least once) and those who do not. This can then be analysed with logistic regression, chi-square etc. For example, van Dongen, Ruiter, Abraham, and Veldhuizen (2014) looked at whether people continued to donate blood (Yes/No), based on the number of blood donations in the follow-up period – zero is No and any positive number is Yes. Similarly in a related study, Veldhuizen, Atsma, van Dongen, and Kort (2012) looked at stopping blood donation (Yes/No). I have also looked at whether people in Pakistan visited a health professional over a month (visited/did no visit), when it would have been possible to analyse the number of visits across the month (histogram of visits displayed in the second panel of Figure 1; Anwar, Green, Norris, & Bukhari, 2017). In these three studies, logistic regression was used.

In data where there are not large numbers of zeros, winsorizing (where extreme values are re-coded to a lower limit) is a method that can be used for dealing with extreme outlying values (e.g. Keller et al., 2020). Skewness can be dealt with either by log or Box–Cox transformations. A key disadvantage of both log and Box–Cox transformations is that they do not handle zeros naturally (i.e. log(0) = ∞), whereas count distributions clearly do.^1^ There are workarounds for dealing with zero values, but no transformation will spread out a stack of zeros (Atkins & Gallop, 2007). For data that are naturally count data, using count distributions also makes more sense than transformation.

Another solution is to switch to non-parametric analyses (e.g. treating the data as ordinal and using Mann–Whitney instead of a t-test, or a Spearman instead of Pearson correlation). However, non-parametric analyses are more limited in their ability to model data. For example, of the classic non-parametric tests, there are none that can account for covariates or nested data. Secondly, the issue here is not that the data is not parametric, but that it is not accurately parameterised in terms of the normal (Gaussian/bell curve) distribution – described by the parameters mean and standard deviation. Instead, there are other distributions – described by different parameters – that produce appropriate analyses. By moving to one of these (count) distributions, essentially any data structure that can be analysed with normally distributed data can be analysed. Finally, this advantage is not merely academic. Not appropriately modelling count data as count data can obscure true relationships. In the Staunton et al. YouTube data discussed here, there are no effects found using linear regression.

In the next section, we will discuss distributions of count variables – non-negative integers. Count variables, though discrete, should not be confused with ordinal variables – counts are an absolute ratio scale (see Williams, 2019 for a useful discussion). That is, they have a meaningful zero value, and eight is meaningfully twice as many as four, for example. This information is also lost changing to classic non-parametric tests. In the discussion, I note that Gamma regression may be used for positive dependent variables with decimals. Especially with self-report, some continuous variables may resemble count variables. For example, ‘minutes of moderate-to-vigorous’ physical activity is often rounded to whole minutes. However, in common with a true discrete count, negative values are not possible.

## Count distributions

The Poisson and negative binomial are discrete probability distributions, both with a lower bound at 0. This property is important, as for most of the dependent variables we are attempting to model here, negative values are not possible – in contrast to classic linear models like regression, where the outcome variable can easily have negative values. A person cannot smoke minus three cigarettes in a day or visit the gym less than no times in a week. Formally, negative binomial is the count of the number of trials in a series of independent and identically distributed binary trials until a specified number of successes (or failures) occurs, and the Poisson is the count of times an event occurs in a period of time. Both distributions assume each event is independent; however, because people change their behaviour based on recent experiences, this may not always be true (Hassan & Bilal, 2008). For example, a person’s preference for choosing to exercise might change based on recent experience. In practice, it may be hard to determine whether observations are truly independent, and this is not further considered in many published applications. The ‘quasi’ distributions discussed later in this section are free of this assumption.

**Figure 2.**
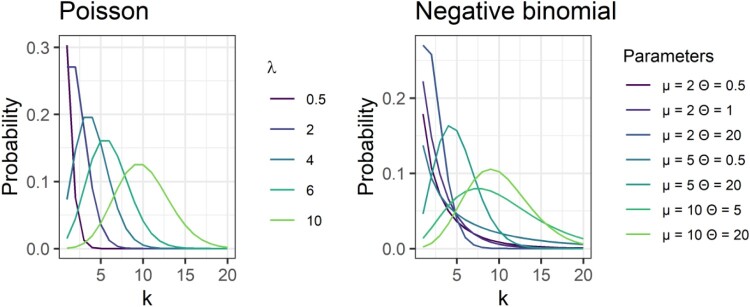
Left panel shows the shape of the Poisson distribution (number of events on the *x*-axis; probability of observing that number of events on the *y*-axis) for various values of *λ*. Right panel shows the shape of the negative binomial distribution for various values of *μ* and *θ*.

The Poisson distribution has a single parameter *λ*, in place of the Gaussian distribution’s *μ* (mean) and *σ* (standard deviation). *λ* is the mean of the distribution. As can be seen in Figure 2, at small values of *λ*, high numbers of zeros are expected, but with a somewhat long tail to higher numbers. Counter-intuitively, for those of us who have always lived and breathed the normal/Gaussian’s mean and standard deviation, the variance of the Poisson distribution is equal to the mean and shares the same parameter *λ*.

As just noted, the tail towards higher numbers is not all that long, so the Poisson distribution might model the number of blood donations in a year, but perform more poorly with the number of cigarettes smoked in a day. This long-tailed property can be caused by over-dispersion, where the spread of values is greater than expected by the distribution. There are several alternative ways to model count data that deal with overdispersion.

The quasi-Poisson model de-links the variance and expected value. The expected value continues to be *λ*, but an additional parameter *ϕ* changing the variance (analogous to the standard deviation in the normal distribution) is then estimated from the data, which can produce larger or smaller amounts of dispersion.

The negative binomial distribution also models greater dispersion than Poisson. Like the quasi-Poisson, it has two parameters, allowing it to accommodate a wider range of distribution shapes than Poisson. It can be parameterised in terms of a mean μ and shape parameter *θ*, where larger values of *θ* indicate greater dispersion.^2^ As can be seen in the right-hand panel of Figure 2, this can better resemble the shape of some of our example variables. While Poisson and negative binomials are both good for modelling highly skewed data, as their parameters increase, the distribution becomes more similar to the symmetrical normal distribution. For Poisson, this happens when *λ* is around 10 (left panel of Figure 2). The Poisson distribution can also be considered a specific instance of the negative binomial, when the dispersion parameter is equal to one(meaning that the mean and variance are equal).

There is also a quasi-negative binomial – but this is only implemented in SAS (in NLMIXED). This is a pity, as the distribution has the type of properties we would be interested in. Specifically, where there are not an oversupply of zeros it can often produce a good fit for more highly skewed data as it has a ‘fat tail’ – more extreme high values^3^ (Hassan & Bilal, 2008; Li, Yang, Famoye, Lee, & Black, 2011).

As a final^4^ distribution for modelling skewed data, the quasi-binomial^5^ (Consul, 1990) can also be useful. It does not model excess zeros, however, but the zero-inflated quasi-binomial can. Similar to the quasi-negative binomial, the quasi-binomial does not assume that the probability of success is constant between trials.

## Zero-inflation and hurdle models

**Figure 3.**
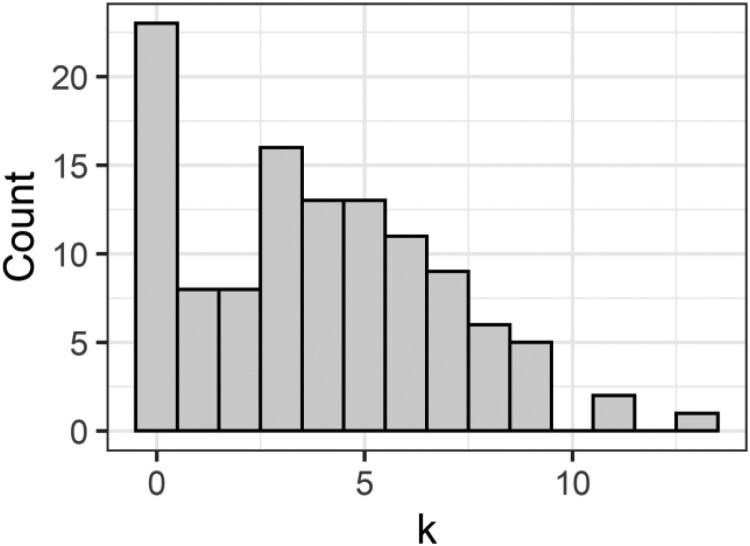
Hypothetical data with a clear bi-modal distribution, with the first peak at zero.

Despite the intuitive appeal inherent in the name ‘zero-inflation’ (e.g. zero-inflated Poisson), the Poisson and negative binomial families can still deal with large numbers of values being zero (Warton, 2005; Xie et al., 2013). However, this is contingent on the mean being relatively low. As an extreme example, in Figure 3 below (which could be standard drinks consumed in an evening, or cigarettes smoked), there is both a peak at zero, and a second peak above zero.

Both zero-inflation and hurdle models are two-step models. They use a first step to model additional zeros – either engaging in the behaviour or not. And then a second Poisson or negative binomial model for modelling the level of engaging in the behaviour. The key difference between zero-inflation and hurdle models, is that in a zero-inflated model, there are two reasons for a participant to score zeros. One is that they don’t engage in the behaviour ever (i.e. a non-drinker), and the second is that a participant didn’t drink in the time period. In contrast, a hurdle model assumes that zeros have a separate cause (e.g. perhaps being a non-smoker), but that if you are a smoker, you won’t have a day where you forget to smoke a cigarette. Social smokers cloud this issue, however, and for this type of reason, it is perhaps unlikely that hurdle models make sense for health psychology. A person that usually engages in vigorous physical activity may have the flu or be busy at work, and therefore score zero, overlapping with people who have routinely sedentary patterns.

## Example use of count distributions

Poisson and negative binomial models have been used across a wide range of fields. Some of the earliest modelling and development was looking at whether some factory workers (in high explosive shell factories!) were more prone to having multiple accidents (Greenwood & Yule, 1920). They are widely used in econometrics, including applications to health (see e.g. Cameron & Trivedi, 2005) and in ecology (see e.g. Vincent & Haworth, 1983).

There are numerous specific examples in the broader field of health. For example, the negative binomial model has been used to examine the effect of different medicines on the number of incontinence episodes over three days (Martina, Kay, van Maanen, & Ridder, 2015). Length of hospital stay is another outcome variable that has been successfully modelled with the negative binomial, which outperformed Poisson (Carter & Potts, 2014). Quasi-binomial was used to test the association between smoking and periodontitis (Zeng et al., 2014). In professional rugby players, coping related to eliciting support was linked to minutes played at a professional level five years later using zero-inflated binomial regression (Rumbold, Fletcher, & Daniels, 2020).

Within health psychology, count models are most often used for modelling longitudinal daily data. For example, Inauen, Shrout, Bolger, Stadler, and Scholz (2016) used negative binomial to model snack consumption within three hour reporting periods (snack counts ranging from zero to four) with generalised estimating equation (GEE) methods. Similarly, Lüscher, Hohl, Knoll, and Scholz (2019) modelled daily number of cigarettes smoked with the negative binomial and zero-inflation within a generalised linear mixed model.

## Poisson and negative binomial regression

The types of analyses we will be examining next are generalised linear models (Nelder & Wedderburn, 1972), extensions of the general linear model (regression, ANOVA etc.) – and seem very much like (linear) regression. In R, they are constructed almost identically to a standard regression model; whereas in SPSS they are accessed through a separate menu that functions quite differently.^6^

A potential key ingredient in any of these regression models is what is described as an *exposure variable* – the time, space or area in which the counts are recorded. If all the counts have the same exposure window (e.g. number of cigarettes for each participant *in the last week*), then there is no need to control for exposure, but in some designs, it may be necessary. In the first example analysis, each video on YouTube has been ‘exposed’ for viewing for the time since it was published. A video that was published two years ago will, all else being equal, have been viewed more times than one published three months ago. Or if the unit of analysis was a family unit, then *family size* might function in the same way. In ecological examples, it is often the size of the hive/colony/breeding site.

Model fit – determining which count distribution to use – can be assessed in a number of ways. Most commonly, fit metrics, particularly AIC are used. AIC balances how well the model fits with the simplicity of the model, with lower values indicating a better model.^7^ Because models with more predictors will tend to provide a better fit, AIC penalises models with more predictors. However, following Buja et al. (2009) and David Fletcher (personal communication, 2015), visual inspection can be a powerful model diagnostic here, in the same way, that visual inspection of a funnel plot often works better than formal tests in meta-analysis (Higgins et al., 2019). To do this, I have written a small R-package (*simfit*), linked in the supplementary materials (https://osf.io/4mjhq/), which shows whether simulating data from the fitted model produces data that resembles the original data. It is possible to see whether too many or too few zeros are produced, relative to the original. Similarly, for the more extreme values, are they extreme enough or too extreme?

**Figure 4.**
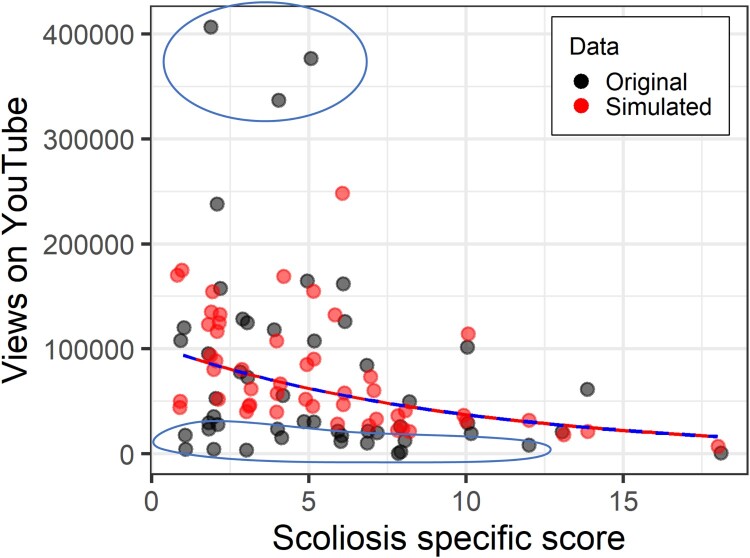
Scoliosis-specific score against number of YouTube views controlling for age. Original data in black, data simulated with a Poisson model in red. Upper and lower ellipses highlight original data points outside the simulated range. Note that Figures from the *sim.plot* function automatically ‘jitter’ data points a little bit so that points that might otherwise be on top of each other are slightly offset.

In Figure 4, for example, the simulated data plotted in red have approximately half the maximum value of the original data, plotted in black. And close to the x-axis, the red points do not go as close to the axis, suggesting there are insufficient low/zero scores. As a model diagnostic, this would then suggest a poor fit.If confirmed in further simulations, different models should be considered, until the distribution of points more similarly map onto each other.

**Figure 5.**
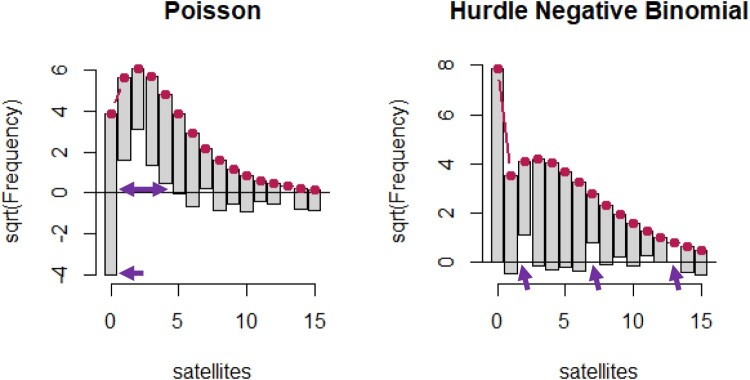
Two example rootograms from the countreg package. The left shows a model fit with a Poisson, and the right, a hurdle negative binomial model. Purple arrows on the left highlight a substantial underprediction of zeros in the Poisson, and then a ‘run’ of over-predictions at one through five. In contrast, the hurdle negative binomial accurately predicts the right number of zeros, and there are only a number of minor deviations (purple arrows), but they are not consecutive.

Unfortunately, it is not currently possible to simulate data for all count models. In addition to using AIC, another visual assessment of fit which is available for some models is Tukey’s (1977) Rootogram,^8^ which is implemented in the package *countreg* (Kleiber & Zeileis, 2016). The expected values for the model are plotted as a red line, and the observed frequencies are plotted as bars, hanging under the curve for the expected values. This hanging style was preferred by Tukey to highlight the deviations along the x-axis. The y-axis is plotted as the square-root of frequencies, to highlight deviations for small observed/expected frequencies. Where the bar goes under the x-axis, the model underpredicts that value, and where the bar does not meet the x-axis, the model overpredicts. In terms of assessing fit, Kleiber and Zeileis highlight two key elements to note. The first is to assess whether the expected number of zeros matches the observed data. The second is the extent to which there are ‘runs’ of successive bars missing the *x*-axis. Two example rootograms are depicted in Figure 5. On the left, the Poisson model clearly underpredicts zeros (hanging all the way down to 4), and there is a run of over overprediction at values 1–4 (peaking at 2). In contrast, the hurdle negative binomial is a better fit, showing little expected/observed discrepancies for the number of zeros, and no clear ‘runs’, with much smaller under and overs along the axis.

A final technical note on count regressions. The models return coefficients that can be interpreted in the same way as a linear model (i.e. *y* = *b*_0_ + *b*_1_*X*_1_, etc.), except that the predicted values are returned as their natural log (i.e. ln($\hat{\mu }$) = *b*_0_ + *b*_1_*X*_1_, etc.). This is similar to how logistic regression returns log odds, which are then transformed (exponentiated) into Odds Ratios. The intercept in untransformed values is *e*^b0^, and the multiplicative effect of a one-step increase in *X*_1_ is *e*^b1^. A more detailed explanation is available in Coxe, West, and Aiken (2009). Practical worked examples are provided later.

## Software choice

Provided in the supplementary materials (https://osf.io/4mjhq/) are the R scripts for all the analyses and figures in this paper, written in R (R Core Team, 2020) in markdown intended for RStudio (RStudio Team, 2020). There are also scripts/demonstrations of all the similar analyses in JAMOVI (The jamovi project, 2021) using the GAMLj module (Gallucci, 2019), and for SPSS (V26, IBM Corp., 2019). Not all are available in those packages (see Table 1), and from my experience, the model diagnostics and comparisons are better in R. However, for a person unfamiliar with R, they may still present a good option for modelling count data.

**Table 1.** Availability of different analyses for count data in different software packages.

	R	SPSS	Jamovi
Poisson	glm(family = ‘poisson’)	Generalised Linear Models (Type of Model = Poisson loglinear)	Yes
Quasi-Poisson	glm(family = ‘quasi-poisson’)	Yes^a^	Yes
Negative binomial	glm.nb	Generalised Linear Models (Type of Model = Custom^b^)	Yes
Quasi-binomial	glm(family = ‘quasi-binomial’)	No	No
Plot-based diagnostics	Yes	No	No
Zero-inflated Models	zeroinfl (dist = ‘poisson’/'negbin')	No^c^	No
Hurdle Models	hurdle(dist = 'poisson'/‘negbin’)	No	No

^a^ You can achieve something similar by changing the ‘Scale Parameter Method’ from 1 (i.e. mean and variance are constrained to being the same) to (Pearson chi-square).

^b^ Do not select ‘Negative binomial with log link’(!).

^c^ Technically you can, but by using R within SPSS, after installing R and integrating it with SPSS. I have done this previously, and found it to be probably more difficult than familiarising yourself with R. Since then R has become easier to use, including an ability to seamlessly read in SPSS data with the *haven* package (Wickham & Miller, 2020).

## Ethics statement

The data from Staunton et al. (2015) did not involve human participants but was based on publicly available data. For Anwar et al. (2017) ethical approval was received from the University of the Punjab (HEC/UCP/1916A) in Pakistan, and we also received approval from the University of Otago Human Ethics Committee (F12/008).

## Analysis walk-through

### Example of highly skewed data

For this analysis walk-through, we will use some data from a project that I was previously involved in, which looked at the popularity versus accuracy of YouTube videos for scoliosis (Staunton, Baker, Green, & Devitt, 2015). The data, and R-code, as well as parallel analyses in Jamovi and SPSS are available at https://osf.io/4mjhq/. The primary outcome is the number of views on Youtube: some of the very popular videos have hundreds of thousands views, whereas some have been viewed by only a handful. This particular dataset does not have a large number of zeros – in fact there are no zeros – but it is a true count distribution, and clearly skewed (see left panel of Figure 1).

In this simple dataset, we will focus on the number of views for each video. As noted earlier, the age of the video serves as an *exposure variable*, which we essentially control for, in not too dissimilar to the way that, for example, socio-economic status or age might be controlled for in a linear regression. The predictors are quality assessments of the videos; the one focussed on here is an ordinal measure of quality, the scoliosis-specific score.

**Figure 6.**
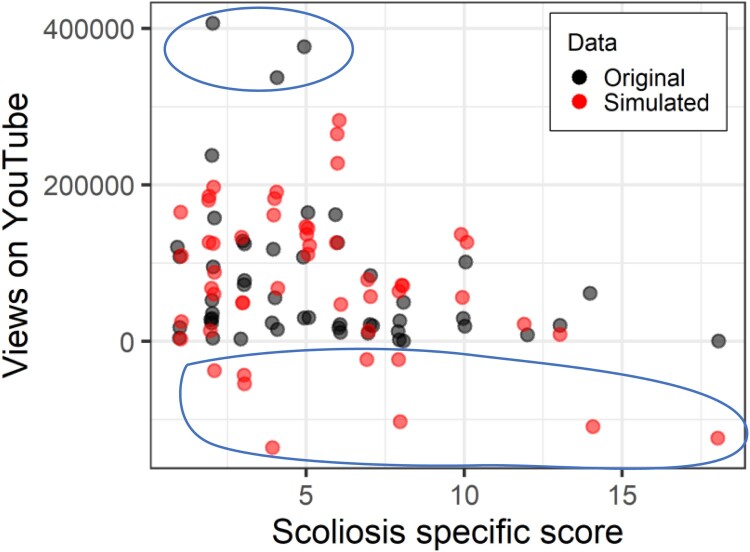
Scoliosis-specific score against number of YouTube views controlling for age. Original data in black, data simulated with a linear model in red. Ellipses highlight where the simulated data does not overlap with the original data.

Firstly, to illustrate why choosing a count model is appropriate, we will fit a traditional linear regression model. The covariate *video age* is significant, indicating that (shockingly) videos that were uploaded longer ago have more views, but the scoliosis-specific quality score is unrelated to the number of views. More interesting is to look at the data simulated fromthe model (Figure 6). Firstly, it doesn’t match the distribution wellin terms of anticipating very high scores, but more importantly, the linear model predicts several quite large values for *negative* views, and obviously, a video cannot be viewed −200,000 times. Count models can never predict values below zero, and certainly not such wildly erroneous ones.

**Figure 7.**
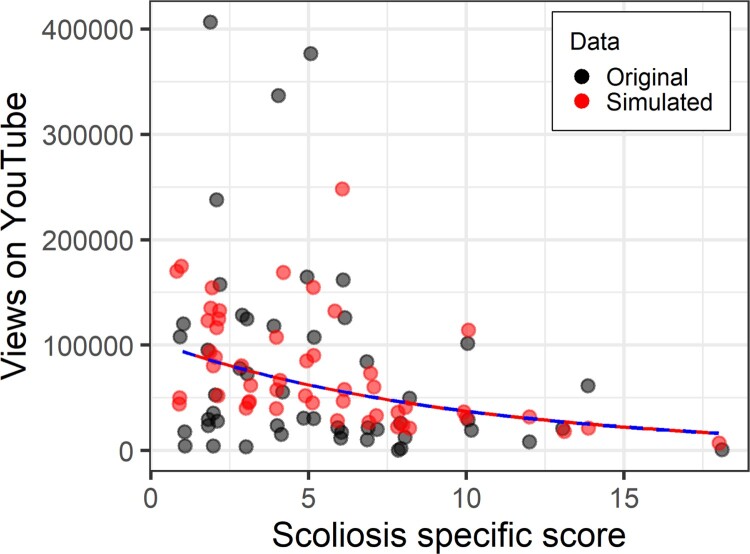
Scoliosis-specific score against number of YouTube views controlling for age. Original data in black, data simulated with a Poisson model in red.

We next fit a Poisson regression to the same data. This time, not only is the video age covariate significant, but there is a relationship between the scoliosis-specific quality score and the number of views of the video (Figure 7) – unfortunately, the more accurate the information in the video, the fewer views. So here we immediately see the potential benefit of fitting a more accurate model. We have been able to detect a likely true effect that was not found in the linear model. The AIC for the Poisson model is 2,761,005.^9^ Looking at the simulated versus original data in Figure 7, it is obvious compared to Figure 6 that there are no simulated values for the number of views below zero. The shape of the simulated data is broadly correct but is not sufficiently dispersed – not enough high values, and not enough low values.To be clear, Poisson is a better choice than linear regression because of fit and appropriateness, not because of predictor significance.

**Figure 8.**
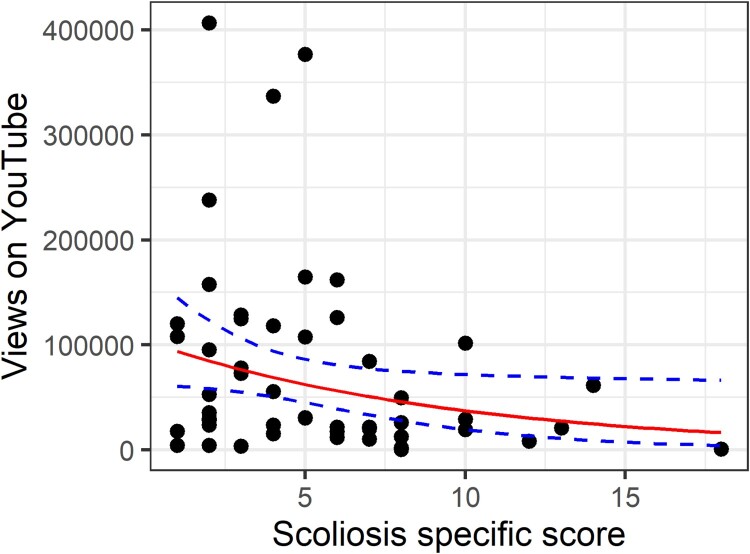
Fit curve for quasi-Poisson (estimated fit in red, 95% CI in blue).

We next fit the quasi-Poisson, which relaxes the constraint that the variance must equal the mean. It is difficult to directly compare quasi-Poisson fit, as neither data simulation, AIC nor rootogram are implemented. However, we can compare the dispersion parameters – set at 1 for the standard Poisson (i.e. mean = variance) and at 71,372 for the quasi-Poisson. The freely varying estimate is not close to 1 (at all!), so it seems that this is likely a much better fit. The fit curve is identical, but the confidence interval estimates are different to the Poisson (Figure 8).

**Figure 9.**
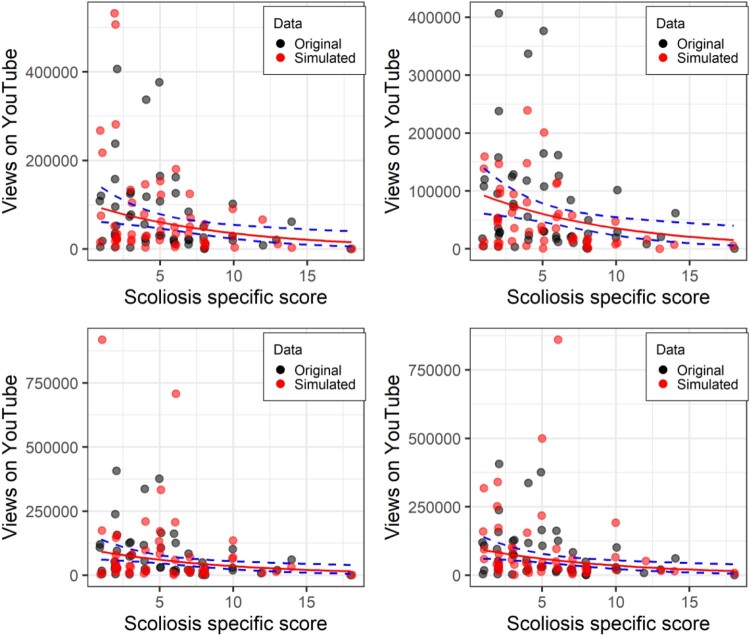
Scoliosis-specific score against number of YouTube views controlling for age. Original data in black, data simulated with a negative binomial model in red. The top-left panel has the random seed set to 45. The subsequent three panels are re-running the same function immediately afterwards, and demonstrates the importance of running multiple simulations.

Next, we move to the negative binomial model. Again, both the age covariate, and the scoliosis-specific score are significant. This time we can compare the AIC for the negative binomial with the Poisson, and it is improved to 1205. And as can be seen in the panels of Figure 9, the simulated data in red better approximates the real data, with more low scores close to the *x*-axis, and more over-dispersion. However, these four panels also clearly illustrate that the simulated data are based on a random process. These are four consecutive runs of the function and produce quite different simulated datasets. Therefore, simulating multiple datasets and comparing them to the observed data is more robust than a single simulation. In future versions of *simfit,* I plan to implement multiple simulations of data automatically. Currently, this can be achieved by either setting the random number seed to different values, or re-running the *sim.plot* function several times. Rootograms appear not to work with count data with such large values (see tutorial files for uninterpretable rootograms).

Finally, because there are no zeros in this dataset, neither zero-inflation or hurdle models make sense. To recap then, the fit and estimates are summarised in Table 2. Both AIC and the simulated data suggest that the negative binomial model is best. The estimated coefficients for the intercept, video age, and scoliosis score are similar for all three count models (the standard linear regression looks different because it is not predicting natural log values). The fitted negative binomial then is:

$$ln(\hat{\mu })=8.79+0.0011(days\;old)+-0.106(scoliosis\;score)$$

**Table 2.** Regression coefficients and model fit for different distribution models.

	Standard linear regression	Poisson regression	Quasi-Poisson regression	Negative binomial regression
Intercept	−85051.90	9.22	9.22	8.79
Standard error	57755.15	0.003	0.692	0.656
* p* value	0.147	<0.001	<0.001	<0.001
Age of video	77.58	0.000977	0.000977	0.00115
* *Standard error	21.21	<0.001	0.0002	0.037
* p* value	<0.001	<0.001	<0.001	<0.001
Scoliosis score	−4428.20	−0.102	−0.102	−0.106
* *Standard error	3185.31	0.001	0.049	0.0002
* p* value	0.171	<0.001	0.045	0.004
AIC	[not comparable]	2761005	NA	1205

Note: Coefficients for linear regression are in number of views. For the count distribution, they are natural logs of the number of views.

The average age of the videos was 2383 (about 6.5 years), and the average scoliosis-specific score 5.38. Substituting those values into the equation (8.79 + 0.0011*2383 + −0.106*5.38) yields a predicted value of 10.84, which exponentiated (*e*^10.84^) is 51,021 views (the average views for a video of average age and score). To understand the multiplicative effect of the coefficient for the scoliosis score (or any other predictor) is to exponentiate the coefficient (here *e*^−0.106^ = 0.90). So for each one-point increase in video quality, as assessed by the scoliosis-specific score, views decrease by 0.90 times (or decrease by 10%).

### Example with a large number of zeros

Because the most common use of count models is to deal with data with large numbers of zeros, the second example has a more ‘usual’ count distribution (right panel of Figure 1), with a clear spike at zero, and a very strong right skew. The maximum count observed is much smaller, here only ten, which is again, more common in many examples of count outcomes.

This data comes from Anwar et al. (2017) with the outcome variable (number of visits to a health professional in 30 days) originally dichotomised into visited or not visited, and modelled with logistic regression. Instead here, we will treat it as the count variable it is. The key predictor is the number of days on which symptoms were reported across the 30 days. This was positively associated with visiting a healthcare professional in the published study. This was a much larger project (mostly unpublished), and so that the demonstration models have some additional predictors, I have included two previously unanalysed measures from the dataset^10^: adapted (and translated) for Pakistan versions of the Beliefs about Medicines Questionnaire General and Specific sub-scales (Horne, Weinman, & Hankins, 1999) and the short form of the Barratt Impulsiveness Scale (Spinella, 2007).

**Figure 10.**
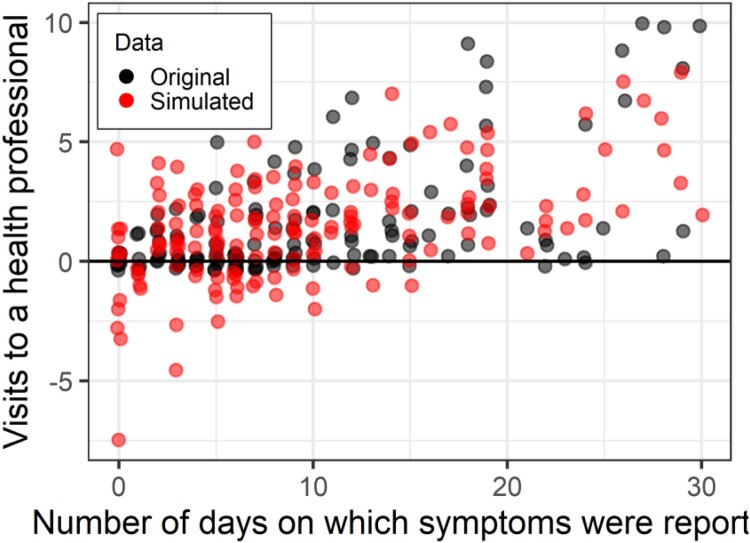
Number of days on which participants reported symptoms as a predictor of days on which participants visited a healthcare professional. Original data in black, Data simulated from a linear model in red.

The data simulated from the linear model (Figure 10) predicts a large number of negative values for visits to a health professional. Further, there is too much dispersion with low numbers of symptom days, and insufficient at a higher number of symptom days.

**Figure 11.**
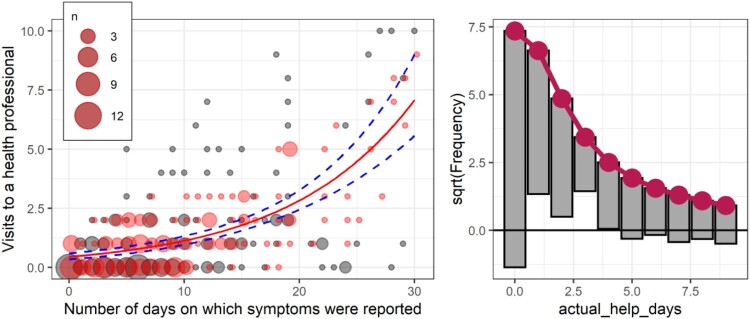
Simulated versus original data with Poisson fit on a scatterplot (left) and rootogram (right). Circle size is used to illustrate the number of overplotted points.

However, the simulated data from the Poisson model is more accurate, although there is less variability in the simulated data (left panel, Figure 11), and as the rootgram shows, there are also too few zeros expected in the Poisson compared to the data (the under hanging value for 0, right panel Figure 11), and a ‘run’ of overprediction for 1–3.

**Figure 12.**
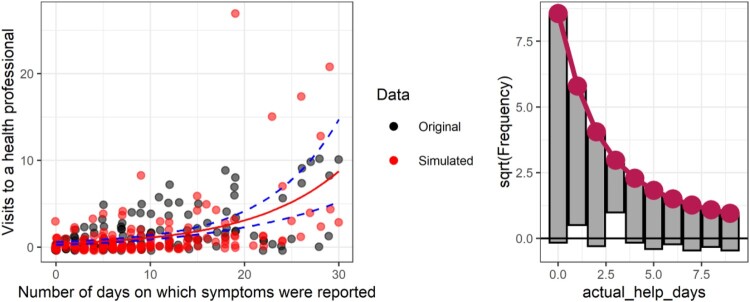
Simulated versus original data with negative binomial fit on a scatterplot (left) and rootogram (right).

The negative binomial shows a much better fit on the rootogram (right panel of Figure 12), but the simulated data shows that the negative binomial fits a handful of unrealistically extreme values. This pattern was largely repeated across several simulations (see tutorial files).

**Figure 13.**
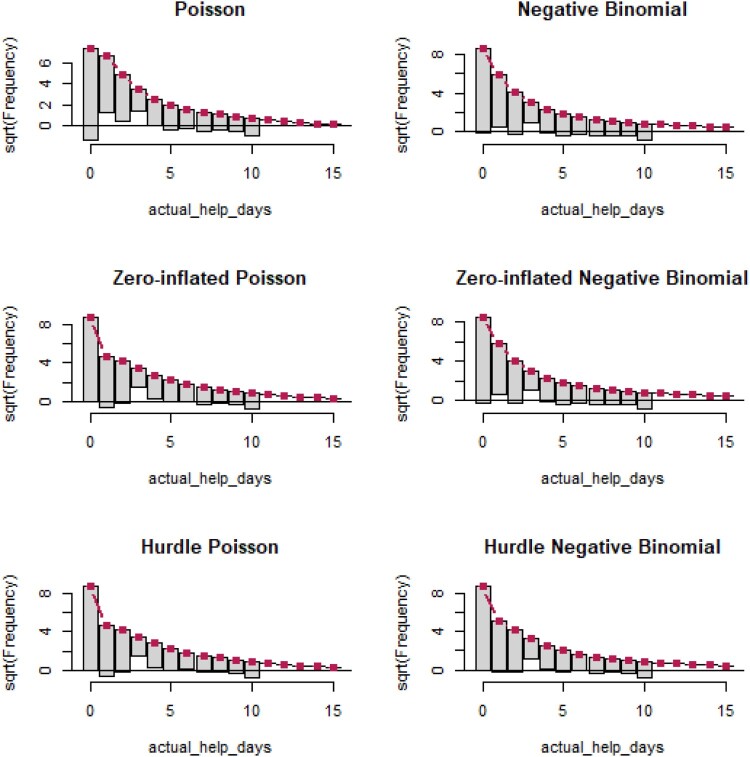
Rootograms for various count models for the number of days on which a health professional was visited.

Because both these models appear like they could be improved upon, and especially because the Poisson is struggling with the number of zeros in the data, trying zero-inflation and hurdle models makes sense here. Simulation is not available for these models, but we can use the rootogram and AIC to compare their fit. Looking at the rootograms across all six models (Figure 13), none of these models seem bad, especially compared to the examples from the *countreg* package (Figure 5). As noted earlier, the Poisson is weak, and probably the weakest, but there is not a lot between the remainder. Based on AIC (Table 3), the zero-inflated negative binomial performs slightly better than the negative binomial. Ultimately, theoretical considerations, and the examination of the predictors for the zero-inflated logistic regression might guide the ultimate model choice.^11^

**Table 3.** Regression coefficients and model fit for different distribution models.

	Poisson regression	Negative binomial regression	Zero-inflated Poisson	Zero-inflated negative binomial	Hurdle Poisson	Hurdle negative binomial
**Intercept**	**−1.17**	**−1.18**	**0.13**	**−1.01**	**0.16**	**−0.04**
* *Std. error	0.35	0.54	0.39	0.50	0.40	0.59
* p* value	<0.001	0.03	0.7	0.046	0.7	0.9
**Symptom days**	**0.092**	**0.104**	**0.069**	**0.107**	**0.067**	**0.077**
* *Std. Error	0.007	0.014	0.009	0.014	0.009	0.015
* p* value	<0.001	<0.001	<0.001	0.017	<0.001	<0.001
**Impulsivity**	**−.012**	**−.014**	**−0.015**	**−0.017**	**−0.015**	**−0.019**
* *Std. Error	0.004	0.007	0.004	0.006	0.005	0.007
* p* value	0.006	0.053	<0.001	0.009	<0.001	0.004
**AIC**	**514**	**458**	**467**	**454**	**467**	**459**

Notes: Estimates are omitted for the zero-inflated and hurdle step of the model for simplicity. These are included in the tutorial files.

For the zero-inflated and hurdle models, it is also not necessary to have the same predictors for the two steps. For example, in the tutorial walk-through, there is a model with only number of symptom days for the zero-inflated/hurdle step, and then a larger model for the count distribution. This simplification actually increases the AIC, so those specific models did not work in this instance, but could be worth exploring for a different dataset.

## Discussion

In this tutorial, two example analyses were presented, one analysing an outcome variable with a large number of zeros and high skewness/dispersion; the second involving high skewness/dispersion. In both cases, using a count distribution to model the outcome variable was clearly more successful than conventional linear models. Further, especially in the implementation in R, using models such as Poisson or negative binomial are clearly analogous to conventional regression models, both in terms of how they are specified, and how the results are presented, so they do not present a challenging shift in terms of learning new analyses. In the second example, it was also clear that models without zero-inflation can still perform well modelling quite substantial numbers of zeros, but there are also times when zero-inflation or a hurdle model might produce a better fit.

The methods presented here are necessarily a subset of the available methods, but should be suitable for a wide range of designs, and are adaptable to the most common ‘standard’ designs. That is, they can easily replace the general linear model: t-tests, ANOVA, regression, and ANCOVA. For those looking for a more technical, but still accessible, account of generalised linear models implementing Poisson and negative binomial, consider Coxe et al. (2009).

As noted earlier, it is also possible to extend generalised linear models into even more complex designs such as longitudinal Ecological Momentary Assessment (EMA)/Experience Sampling Method (ESM) data with generalised linear mixed models (GLMMs) and generalised estimating equations (GEEs). However, while incorporating count distributions into these methods represents some improvement, some recent work suggests that these do not adequately model EMA/ESM data (Hasselman & Bosman, 2020). Repeated observations over time within an individual are clearly not independent, but traditional linear methods tend to model influence only from the previous observation (a lag of 1 in autoregression), whereas human memory can provide influences from weeks or months ago (Hasselman & Bosman, 2020; Olthof, Hasselman, & Lichtwarck-Aschoff, 2020). Similarly, for behaviour change, we would expect to see potentially marked abrupt change, change in variability, and other phenomena not modelled within linear methods. For a health psychology-based expositionof this see Heino, Knittle, Noone, Hasselman, and Hankonen (2020) and accompanying supplementary materials.

Cameron and Trivedi (2013) cover more alternative models for count data, including for longitudinal data. It is also possible to use Bayesian estimation, which may have particular benefits in more complex models with multiple levels of nesting (personal communication, David Fletcher, Matthew Schofield, 2015). I have not touched on truncated Poisson/negative binomial regression. These fit distributions that have *no* zeros, but otherwise display count properties. The most obvious examples are where a sample is recruited in a place where people are actively engaging in the behaviour being measured (last 30-day gym visits in a sample of people recruited *at the gym*, last 12-month blood donations recruited *at blood donation sites*, or length of hospital stay), or where the exclusion criteria exclude zero values (number of standard drinks consumed at the last party attended, and data from those not drinking is excluded). The *svyglm* function in the *survey* package in R (Lumley, 2020) facilitates survey weighting for some types of count regression. Atkins and Gallop (2007) also describe how dyadic data can be modelled with counts.

For continuous, skewed dependent variables, treating them as a quasi-count is not the only or best option. The Gamma distribution, also within the generalised linear model family, has a similar shape to Poisson andnegative binomial but is naturally continuous (Ng & Cribbie, 2017). Gamma regression is implemented in R in a very similar way to count regression.

Limitations of count regression include the need to transform coefficients to make them interpretable. However, this is also a feature of alternate transformations such as log and box-cox. The implementation of different distributions and analyses is not consistent across software packages, and my preferred visual inspection for fit is not available for every type of analysis here. There is also a risk of overfitting. This can occur by putting too much weight on a single or small number of simulations for the visual inspection method. However, there is a wider risk of overfitting, in that the observed data is a single sample, and that tailoring the model too closely to the observed data risks fitting to random variation rather than the underlying process.

Count distributions successfully model discrete outcome variables that have either a large number of zeros and/or are strongly skewed. Moreover, for true count variables, these distributions are more technically accurate, with models never predicting values below zero, which would never be possible for the outcome variable. Further, relative to the common alternatives, such as using non-parametric tests, modelling the outcomes using these distributions is more powerful and flexible.

## Notes

1 Although I am discussing count distributions here, it is also worth noting that very small values just above 0 (e.g., 0.001) become very large and influential values when logged. So logging a continuous variable with very small values will also not perform well.

2 The negative binomial is a distribution developed to model a series of independent and identically distributed trials with a binary outcome, with a probability of success (*p*), before a set number of failures (*r*) occurs. Here, I have described an alternative parameterisation which better describes the *shape* of the distribution, rather than its underlying mechanism. It also is more obviously analogous with the normal and Poisson distribution described as *μ* and *θ*.

3 Student’s *t*-distribution is probably the most well-known fat-tailed distribution. As the sample size/degrees of freedom increases, the distribution converges tothe normal distribution. But with smaller samples, the tails fatten, meaning that the value of t required to reject the null hypothesis increases well above 1.96, making the t-test more conservative as the sample size decreases.

4 There are of course, more distributions (e.g. the beta-binomial), but in practice, Poisson and negative binomial are the most commonly reported and compared (Zeng et al., 2014).

5 You might note that I have not discussed the binomial distribution, but am jumping straight to the quasi-binomial. The binomial distribution does not have the shape we are interested in here, but the quasi-binomial can sometimes be useful.

6 This includes NOT selecting the ‘negative binomial’ option to conduct a negative binomial regression.

7 AIC estimates how much ‘information’ a model loses, so lower values are indicate better fit.

8 Named, apparently, because the *y*-axis is the square-root of the frequency.

9 AIC is available for linear regression, but the values are not comparable.

10 I have no strong justification, theoretical or otherwise for the choice of these variables. The original design choices would have been that people with more negative attitudes towards medicines might be less likely to seek conventional medical treatment.

11 As this particular example was constructed for this tutorial, I don’t have a clear choice here.
